# Discovery of Novel Pyridin-2-yl Urea Inhibitors Targeting ASK1 Kinase and Its Binding Mode by Absolute Protein–Ligand Binding Free Energy Calculations

**DOI:** 10.3390/ijms26041527

**Published:** 2025-02-12

**Authors:** Lingzhi Wang, Yalei Gao, Yuying Chen, Zhenzhou Tang, Xiao Lin, Meng Bai, Pei Cao, Kai Liu

**Affiliations:** 1 Guangxi Key Laboratory of Marine Drugs, Guangxi University of Chinese Medicine, Nanning 530200, China; wanglingzhi2022@stu.gxtcmu.edu.cn (L.W.); gaoyalei0322@163.com (Y.G.); ah20211209@163.com (Y.C.); tangzz@gxtcmu.edu.cn (Z.T.); linx@gxtcmu.edu.cn (X.L.); xxbai2014@163.com (M.B.); 2 Institute of Marine Drugs, Guangxi University of Chinese Medicine, Nanning 530200, China

**Keywords:** apoptosis signal-regulating kinase 1, pyridin-2-yl urea inhibitors, absolute binding free energy calculation, binding mode prediction

## Abstract

Apoptosis signal-regulating kinase 1 (ASK1), a key component of the mitogen-activated protein kinase (MAPK) cascades, has been identified as a promising therapeutic target owing to its critical role in signal transduction pathways. In this study, we proposed novel pyridin-2-yl urea inhibitors exhibiting favorable physicochemical properties. The potency of these compounds was validated through in vitro protein bioassays. The inhibition (IC_50_) of compound **2** was 1.55 ± 0.27 nM, which was comparable to the known clinical inhibitor, Selonsertib. To further optimize the hit compounds, two possible binding modes were initially predicted by molecular docking. Absolute binding free energy (BFE) calculations based on molecular dynamics simulations further discriminated the binding modes, presenting good tendency with bioassay results. This strategy, underpinned by BFE calculations, has the great potential to expedite the drug discovery process in the targeting of ASK1 kinase.

## 1. Introduction

Mitogen-activated protein kinase (MAPK) cascades play an essential role in stress-responsive signal transduction pathways that participate in many cellular processes mainly through three classes of kinases: MAPKs, MAPK kinases (MAP2Ks) and MAPK kinase kinases (MAP3Ks) [1]. Numerous reports have found that MAPK pathways involved in many cancers and diseases would be attractive targets for therapeutic intervention [2,3,4,5]. These components, including extracellular signal-regulated kinases (ERK1/2), c-Jun amino (N)-terminal kinases (JNK) and p38 from MAPKs, mitogen-activated protein kinase kinase (MEK1/2) from MAP2Ks and the rapidly accelerated fibrosarcoma (RAF) from MAP3Ks, are frequently investigated and targeted in cancer drug discovery [5].

Apoptosis signal-regulating kinase 1 (ASK1), also a key component of MAPKs, has been gradually recognized as an upstream regulator of JNK and p38 isoform signaling pathways. Its activation is closely associated with a diverse range of stressors, including reactive oxygen species (ROS), endoplasmic reticulum (ER) stress, heat shock and osmotic shock [6,7]. Consequently, ASK1 has been explored as a promising therapeutic target for the treatment of numerous diseases.

Several inhibitors specifically targeting ASK1 have been reported [6,8,9,10,11,12,13,14,15,16,17]. Among these drug candidates, Selonsertib, which was developed by Gilead Sciences and is the first clinical ASK1 inhibitor in the trial treatment of pulmonary arterial hypertension (PAH), diabetic kidney disease (DKD) and nonalcoholic steatohepatitis (NASH). However, Selonsertib did not show sufficient evidence of efficacy for PAH [18], DKD [19] or in the two Phase III clinical trials for NASH (NCT03053050, NCT03053063). Although these failures of Selonsertib discourage its exploration as a monotherapy, the clinical trials do suggest that combination therapy with ASK1 inhibitors is attractive [20]. Therefore, continual investigation of the inhibitors targeting ASK1 is still required for the exploration of the role of ASK1 regulation in diseases.

Because the safety and appropriate physicochemical properties of Selonsertib were thoroughly evaluated in clinical trials, further ligand optimization based on Selonsertib has been rightfully proposed. Inspired by the successful replacement of the benzene ring with a non-aromatic ring in Selonsertib [21], we tried to increase the fraction of sp^3^ carbon atoms (Fsp^3^), which is an important drug-like parameter of the inhibitor [22].

Here, we proposed a structural optimization of pyridin-2-yl urea derivatives with a non-aromatic moiety based on a previously established robust synthesis route [23]. The binding mode of the proposed compounds was initially examined through molecular docking. Their binding affinities were subsequently estimated using highly accurate absolute binding free energy calculations based on all-atom molecular dynamics simulations. Finally, the affinity predictions were compared with the results from in vitro bioassay studies. The computational workflow described here provides a practical solution for distinguishing the binding modes of inhibitors, facilitating rational drug design.

## 2. Results

### 2.1. Design of Pyridin-2-yl Urea Derivatives

Previous research indicated that the removal of the isopropyl-triazole from Selonsertib resulted in about a 2000-fold loss of in vitro potency. Therefore, we decided to maintain the isopropyl-triazole fragment in our design. Given that the crystal structure shows compatibility with the oxacyclohexane ring within the binding pocket of ASK1 [21], the pyridin-2-yl urea moiety was proposed and introduced into Selonsertib.

Table 1 shows the selected physicochemical properties of the proposed compounds, including molecular weight, logP, the number of H-bond acceptors (HBAs) and donors (HBDs), polar surface area (PSA), the number of rotatable bonds (ROTBs), the fraction of sp^3^ carbon atoms (Fsp^3^) and quantitative estimation of drug-likeness (QED). Compared with Selonsertib, the relatively higher QED of compounds was predicted, suggesting a good start for the ligand optimization.

### 2.2. Binding Mode Prediction by Molecular Docking

**Figure 1:**
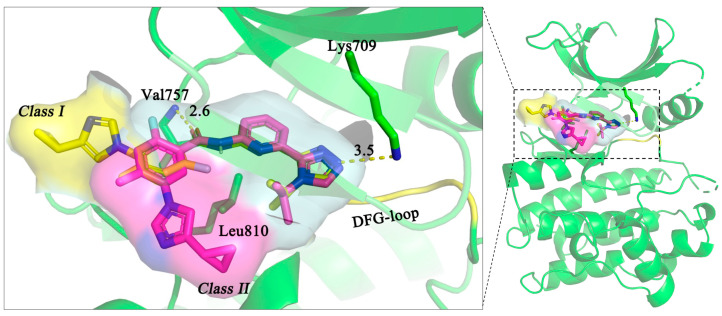
Comparison of binding mode for Selonsertib in ASK1. The binding pose of Selonsertib was from PDB (yellow, 6OYT) and from molecular docking (magenta). The yellow dashed line refers to the H-bond interaction (Å).

In order to further optimize the ligand, the binding mode of our compounds was predicted by modular docking. Figure 1 shows the binding modes of our compounds and compares them with that of Selonsertib, whose structure has been elucidated via crystal structure analysis [12]. As suggested by the crystal structure, key interactions between Selonsertib and the ASK1 pocket mainly include two H-bond interactions between the imidazole and Lys709, the carbonyl group and Val757, and a potential CH-π interaction with the side chain of Leu810. Interestingly, molecular docking also suggests an alternative binding mode (Class II) that is different from the crystal one (Class I). Although the second binding mode shares these similar binding interactions, the imidazole moiety adopts another direction, approaching the DFG-loop (Class II). These two alternative binding modes (Class I and II) were also found for compounds **2**–**7**, which were listed and compared in the Supplementary Information.

### 2.3. Synthesis of Compounds and Their Inhibitory Activities

**Figure 2:**
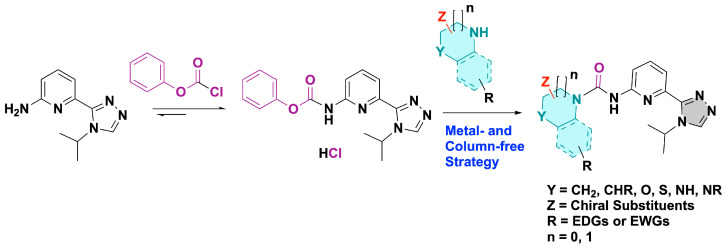
Synthetic route of pyridin-2-yl ureas [23].

Because pyridyl urea is a significant and popular moiety found in various small molecular drugs and candidates, a green and efficient synthesis process for these compounds was strongly pursued [24]. We have previously explored and built an efficient and environmentally friendly protocol for the synthesis of pyridin-2-yl ureas via the phenyl carbonochloridate as an activator, as shown in Figure 2 Following a plausibly concerted mechanism, the corresponding raw products were further recrystallized and purified through a biphasic solvent extraction system of a CHCl_3_/H_2_O mixture [23]. Currently, we extended the reaction to five-membered cyclic amines. Except for the three previously synthesized compounds **1**, **6** and **7**, a similar high yield was also achieved for these five-membered cyclic amines, with an average conversion rate at 91%. This result also highlights the reliability of the synthesis procedures. The detailed NMR data of these compounds are available in the Supplementary Information.

An in vitro bioassay of ligands against the ASK1 protein was carried out, and the details of assay curves are listed in the Supplementary Information. Table 2 compares their inhibitory activities. Compared to the oxacyclohexane in V3S [21], the relatively weaker binding affinity of the smaller pyrrolidine ring of compound **1** suggests that the pocket has a large volume. Thus, compounds **2**–**5**, which contain an indoline ring, had increased IC_50_ values. The methoxy group of compounds **2** and **4** resulted in relatively stronger inhibition, in sharp contrast to the trifluoromethyl group of compounds **3** and **5**. However, the significantly different inhibitory activities of compounds **6** and **7** highlights the importance of substitution position. The IC_50_ of compounds **2**, **4** and **6** were 1.55 ± 0.27, 45.27 ± 4.82 and 2.92 ± 0.28 nM, respectively. 

### 2.4. Binding Affinity of Compounds by BFE Calculation

Although two different docking poses of Selonsertib were predicted by molecular docking, the same docking score was observed. In addition, for compound **6**, which is a strong binder, the docking scores were −8.50 and −8.63 kcal/mol for the two classes of poses. The close values indicated the limited discrimination capability of the docking score. Thus, an accurate binding free energy calculation was performed to discriminate the possible binding mode of these compounds. The BFE calculation indicated that the affinity from Class I was stronger than that of Class II for Selonsertib. This consistency with the result found in the crystal structure suggested that the binding mode was correctly distinguished by the BFE calculation. Encouraged by this discrimination capability from the BFE calculation [25], we further compared the BFE for compounds **2**–**7**. Surprisingly, the BFE calculation suggested that the binding mode of our compounds preferred the docking mode of Class II.

## 3. Discussion

It is a long journey for a compound to become a drug because drug discovery is a generally risky and costly process with high failure rates [26]. Thus, profiling the desired properties of a drug candidate is strongly required for clinical success [27]. The properties listed in Table 1 suggest that these proposed pyridin-2-yl urea compounds possess favorable drug-like characteristics. The relatively higher QED and Fsp^3^ values of these compounds compared to those of Selonsertib indicate a promising starting point for our design. Moreover, considering the highly conserved ATP-binding site of kinases, the introduction of a non-aromatic ring may be further beneficial to the binding site’s selectivity [21]. Therefore, to further explore the binding pocket’s tolerance, we proposed compounds **2**–**7** with the indole and tetrahydroquinoline units.

Understanding the binding mode is essential for elucidating the interactions between ligands and target proteins, which is also crucial for improving their binding affinity and selectivity. Although the binding mode of Selonsertib (Class I) was validated by experimental data, the binding mode of Class II is also highly probable and commonly observed in other kinase inhibitors, such as cyclin-dependent kinase 2 [28], Janus kinases [29] and Bruton tyrosine kinase [30]. It is also noteworthy that similar compounds may adopt different binding modes [31], and even the same inhibitor may exhibit different binding modes for similar targets [32].

Compounds **2**, **4** and **6** show nanomolar inhibitory activity. Their binding modes were compared using docking poses. A similar binding mode of Selonsertib was initially deduced; however, poor correlation was found between the ΔG and docking score of Class I, suggesting different binding modes of these compounds. The docking score may not be accurate for these structurally similar compounds; therefore, the BFE calculation was performed to compare their binding affinities.

The BFE calculation of Selonsertib has successfully identified the favorable binding mode of Class I, which was confirmed by the crystal structure. For other compounds, the discrimination capability of the BFE was also superior to that of the docking scores. Although the BFE calculation failed to predict the affinity, the BFE results presented a good tendency compared to the experimental data.

Determining the inconsistency between the calculated results and the experimental data was not a trivial task. A 5 ns unrestrained molecular dynamics simulation was used to explore the potentially flexible binding conformation and the initial docking pose of complex. The RMSD changes of the protein and ligand were compared in the Supplementary Information. The flat plateaus indicated that the complex became stable during the simulation time. In theory, the restraint force evaluated from these equilibrium regions will finally cancel out [33]. Thus, any snapshot from the equilibrium region would be acceptable. However, the BFE calculation was sensitive to the structure of complex [34]. In our case, the binding mode of Class II was unexpected in advance. Thus, only the final snapshot of the BFE calculation may be not well chosen [35,36]. For the evaluation of the contribution of ELE and vdW, a dense set of 21 and 26 discrete windows were used to guarantee the necessary overlap of neighboring windows. The uncertainty of about 1.0 kcal/mol was also acceptable [37]. However, for some slow equilibration systems, the current settings may not be good enough for adequate sampling [38]. Therefore, further enhanced sampling methods [39,40,41] and optimized λ windows [42] may be necessary for accurate calculations. It should also be noted that the experimental Gibbs free energy was derived based on the IC_50_ instead of the dissociation constant.

**Figure 3:**
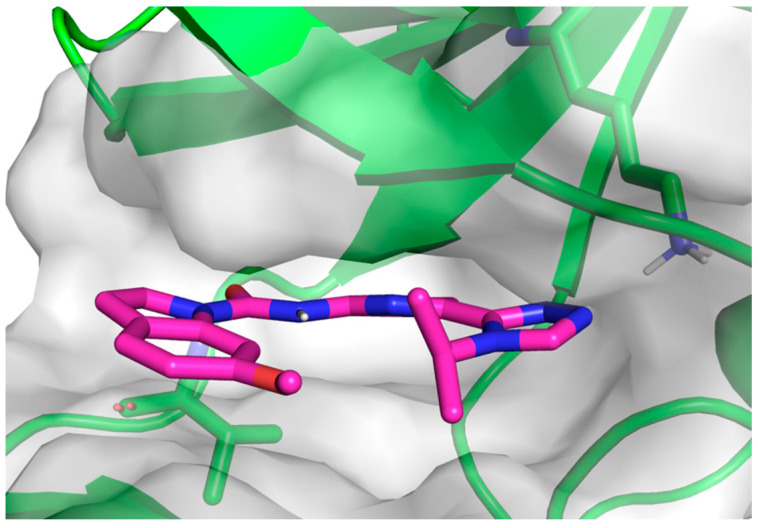
Binding mode of compound **2** after MD simulation. This complex was the final snapshot from the 5 ns MD production simulation.

The binding mode is essential for further ligand optimization. The identification of the binding mode by BFE calculations would be beneficial for the acceleration of the drug discovery process. Figure 3 shows the plausible binding mode of compound **2** predicted by the BFE calculation. The bioassay results suggest that the flat pocket prefers a methoxy group to a trifluoromethyl group. The sensitivity of the binding site also indicates that our optimization strategy to improve the selectivity of the ligands by introducing a non-aromatic moiety into Selonsertib was feasible. Further ligand optimization has also been carried out using this binding mode. For example, macrocyclization of the compound in the binding pocket was a promising strategy for this further design [12,43,44]. Inspired by the work of feline sarcoma-related tyrosine kinase [45], further substitution on the tetrahydroisoquinoline ring of compound **6** was also explored to increase the selectivity.

## 4. Materials and Methods

### 4.1. Pharmaceutical Properties of Pyridin-2-yl Urea Derivatives

The quantitative estimation of drug-like (QED) properties for the proposed compounds was conducted using the RDKit toolkit (v.2020303) [46]. These selected molecular properties, including molecular weight (MW), logP, number of hydrogen bond acceptors (HBAs) and donors (HBDs), topological polar surface area (PSA), the number of rotatable bonds (ROTBs) and the fraction of sp^3^ carbon atoms (Fsp^3^), were calculated.

### 4.2. Molecular Docking

The molecular binding mode was predicted via molecular docking using Glide module (v.9.1) of Schrödinger software [47]. The co-crystal structure of ASK1 complex was retrieved from PDB bank (PDB code: 6XIH). Then, the complex was prepared by protein preparation module of Schrödinger software. Specifically, the protonation states of residues were predicted by the PROPKA toolkit at a pH of 7.0. The grid of docking pocket was then generated using the default crystallographic ligand (V3S) as reference by Glide module. Finally, molecular docking was conducted using Glide standard precision (SP) mode. The ligand was prepared by the ligand preparation modules using all default values.

### 4.3. Binding Free Energy (BFE) Calculation by Molecular Dynamics

Molecular dynamics (MD) simulation was performed using the Particle Mesh Ewald (PME) module of AMBER software (v.2020) [48]. Based on the docking poses of ligand with ASK1 catalytic domain, the complex was solvated in a cubic water box, maintaining a minimal distance of 8.0 Å from system boundaries. Necessary counter ions (Na^+^ or Cl^-^ ions) were added to neutralize the system for simulation. The ff14SB force field and TIP3P water model were applied to the protein and water molecules, respectively, while the GAFF2 force field and RESP charge for the ligand were assigned based on geometry optimization at the HF/6-31G(d) level of theory using Gaussain16 [49].

The stability of complex was initially examined via standard MD simulation, as described in previous papers [25,50]. Briefly, the complex was optimized in 1000 cycles with half of steepest descent and half of conjugate gradient minimization. Subsequently, the complex box was gradually heated to 298 K over 200 ps using an NVT ensemble simulation (constant number of atoms, volume and temperature) and changed to NPT ensemble (constant number of atoms, pressure and temperature) to optimize the solvent environment for 100 ps simulation. During the NVT and NPT processes, the complex was restrained with 0.5 kcal/mol/Å^2^. After that, 500 ps NPT equilibrium simulation was performed without any constraint. Finally, a 5 ns production run was conducted to obtain an equilibrated complex.

For each complex, three parallel independent production runs were performed. The root-mean-square deviations (RMSD) of the heavy atoms in ligand were calculated. If the RMSD exceeded the threshold value of 2.0 Å, the ligand was deemed unstable in binding pocket [51,52]. Otherwise, the final snapshot with the smallest RMSD of ligand was selected as the starting geometry for the subsequent binding free energy (BFE) calculation.

For the BFE calculation, a force constraint was introduced to enhance sampling [33]. Three reference atoms from the protein and ligand were selected based on their fluctuations during MD simulation. The detailed selection of reference atoms is described in previous studies [25]. In the BFE calculation, the contributions were divided into electrostatic (ELE) and van der Waals (vdW) components using a series of discrete windows (λ) based on thermodynamic integration (TI) method. This process was similar to the above general MD simulation, but with different coupling λ values. Firstly, the final snapshot from previous MD run was further optimized using 500 steepest descent cycles followed by 500 conjugate gradient steps. It should be noted that only 1000 steepest descent cycles were performed in the minimization of the vdW term. Next, a 200 ps heating procedure was conducted in NVT ensemble, followed by 500 ps equilibration in the NPT ensemble. Finally, a 10 ns production run was carried out. For the ELE calculation, 21 windows with equal intervals were used (λ = 0.00, 0.05, 0.10, 0.15, 0.20, 0.25, 0.30, 0.35, 0.40, 0.45, 0.50, 0.55, 0.60, 0.65, 0.70, 0.75, 0.80, 0.85, 0.90, 0.95, 1.00), whereas 26 windows were used for vdW calculation (λ = 0.00, 0.04, 0.08, 0.12, 0.16, 0.20, 0.24, 0.28, 0.32, 0.36, 0.40, 0.44, 0.48, 0.52, 0.56, 0.60, 0.64, 0.68, 0.72, 0.76, 0.80, 0.84, 0.88, 0.92, 0.96, 1.00). The final binding affinity was estimated using the TI method from the *alchemlyb* toolkit [53].

### 4.4. General Route of Chemical Synthesis

Compounds were synthesized based on our efficient and environmentally friendly protocol [23]. Initially, we selected the commercially available 6-(4-isopropyl-4H-1,2,4-triazol-3-yl) pyridin-2-amine (IPTPA) as the starting reactant. Subsequently, 1.0 equivalent of phenyl chloroformate was mixed and agitated overnight. Following the addition of aromatic/non-aromatic amines together with N,N-diisopropylethylamine, the desired pyridin-2-yl ureas were obtained with high yield via a concerted mechanism [23].

Aside from compounds **1**, **6** and **7**, which were synthesized previously [23], we successfully synthesized four new pyridin-2-yl urea compounds in this study.

^1^H and ^13^C spectra were collected on 500 MHz NMR spectrometer (Bruker AVANCE). Chemical shifts for protons are reported in parts per million (ppm) downfield and are referenced to residual protium in the NMR solvent (CDCl_3_ = δ 7.26). Chemical shifts for carbon are reported in parts per million downfield and are referenced to the carbon resonances of solvent (CDCl_3_ = δ 77.16). Data are represented as follows: chemical shift, multiplicity (br = broad, s = singlet, d = double, t = triplet, q = quartet, m = multiplet, heptet using full name), coupling constants in Hertz (Hz) and integration.

High-resolution mass spectra (HRMS) were collected using a Waters XEVO G2-S Q-TOF-MS mass spectrometer (Waters Corporation, Boston, MA, USA) in positive mode using MeCN/H_2_O; I Class liquid chromatograph; ACQUITY UPLC HSS T3 C18 (2.1 × 100 mm, 1.8 μm) chromatographic column; and MassLynxV4.2 analysis software.

Analytical thin layer chromatography (TLC) was performed on precoated silica gel GF254 HPTLC plates (5 × 10 cm^2^) purchased from Yantai Jiangyou Chemical Co., Ltd. (Yantai, China). The developed chromatogram was analyzed by UV lamp (254 and 365 nm). The non-UV active compounds were generally visualized through placing the plates in a sealed TLC tank containing iodine and silica gel (the 160–200 mesh mentioned above), if necessary, with the aid of a heating gun. The progress of reaction was also monitored by TLC stained with 5% *v*/*v* ethanol aqueous solution of concentrated H_2_SO_4_ and estimated in a concentration- and time-dependent manner.

Pyrrolidine (99%) was supplied by Shanghai Macklin Biochemical Technology Co., Ltd. (Shanghai, China) Phenyl chloroformate (98%) was purchased from Shanghai Aladdin Biochemical Technology Co., Ltd. (Shanghai, China) 6-(Trifluoromethoxy)indoline (98%), 6-methoxyindoline (98%), 5-(trifluoromethyl)indoline (97%), 1,2,3,4-tetrahydroquinoline (99%), 1,2,3,4-tetrahydroisoquinoline (98%), 6-(4-isopropyl-4H-1,2,4-triazol-3-yl)pyridin-2-amine (IPTPA, 95%) and N,N-diisopropylethylamine (DIPEA, 99%) were obtained from Shanghai Bidepharm. 5-Methoxyindoline (95%) was provided by Shanghai Leyan Co., Ltd., Shanghai, China.

### 4.5. Expression and Purification of ASK1

The catalytic domain of ASK1 (encoding residues 659–951) was modified to include an N-terminal 6XHis tag and cloned into expression vector, pMCSG7. The recombinant human ASK1 protein was expressed in *E. coli* Rosetta2 (DE3) in LB medium, containing 100 µg/mL ampicillin and 34 µg/mL chloramphenicol. A total of 2 mL overnight culture was diluted 100-fold into 200 mL LB and grown at 37 °C with shaking at 200 rpm until the OD_600_ reached 0.8. Subsequently, 0.5 mM IPTG was added, and protein expression was induced overnight at 20 °C.

The pellets were collected by centrifugation and resuspended in HEPES buffer (50 mM HEPES, 300 mM NaCl). After adding ddH_2_O, the suspension was placed on ice and subjected to ultrasonication (3 s pulses with 5 s intervals) for 15 min. The lysate was then separated by centrifugation at 18,000× *g* for 15 min, and the supernatant was purified using Ni-affinity chromatography columns with a wash buffer (50 mM HEPES, 300 mM NaCl and 25 mM imidazole) and an elution buffer of the same composition but containing 250 mM imidazole. The concentration of the purified protein was estimated using a BCA protein assay kit (Solarbio, Beijing, China).

### 4.6. ASK1 Enzyme Inhibition Assay

The IC_50_ value for each inhibitor was determined using the ADP-Glo™ protocol. Inhibitors were prepared as a series of 1:1 serial dilutions (final concentration of 10 μM) in 40 mM HEPES buffer (pH 7.5) containing 20 mM MgCl_2_, 0.1 mg/mL BSA, 50 μM DTT and 5% DMSO.

The kinase assays were conducted in a 5 μL volume containing the following final concentrations: 6.25 ng/μL active ASK1, 25 μM ATP and 0.1 μg/μL MBP. The reaction was terminated by adding 5 μL of ADP-Glo reagent and incubated at room temperature for 40 min. Then, 10 μL of kinase detection reagent was added, and the mixture was incubated for an additional 30 min. Luminescence was measured using a VICTOR Nivo microplate reader (PerkinElmer, Waltham, MA, USA), and the concentration–response curve and half-maximal inhibitory concentration (IC_50_) values were obtained by fitting the data using GraphPad Prism (v.9.0) software.

## 5. Conclusions

Kinases are one of the most significant drug targets in the human proteome due to their crucial regulatory roles in signal transduction pathways. ASK1 has been recognized as a promising therapeutic target because of its critical role in modulating signaling pathways. In this study, we proposed novel pyridin-2-yl urea inhibitors targeting ASK1. The favorable physicochemical properties of these compounds were compared with those of the known inhibitor Selonsertib. The best potency (IC_50_) of these compounds was 1.55 ± 0.27 nM using an in vitro bioassay. To further optimize these ligands, two comparable binding modes were suggested using molecular docking. Therefore, absolute binding free energy calculations based on molecular dynamics simulations were conducted to determine the plausible binding modes. A 485 ns simulation was performed for each ligand. Compared with the in vitro bioassay results, the BFE calculation indicated that the predicted affinity of the second class of the binding mode was qualitatively closer. Although the accuracy of the BFE calculations required further improvement, the BFE shows the great potential to accelerate the drug discovery process targeting ASK1 kinase. Further cell-based in vitro tests and mouse models are under construction to assess the anti-cancer potential of these compounds.

## Figures and Tables

**Table 1:** Selected physicochemical properties of compounds.

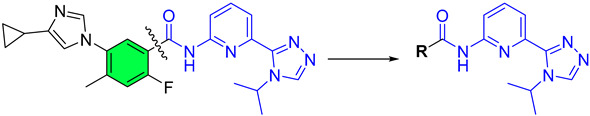									
Compound	R	MW	logP	HBA	HBD	PSA	ROTB	Fsp3	QED
**Selonsertib** [6]	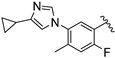	445.5	4.7	5	1	90.5	6	0.3	0.5
**V3S** [21]	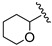	315.4	2.4	5	1	81.9	4	0.5	0.9
**1**	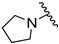	300.4	2.5	4	1	75.9	3	0.5	0.9
**2**	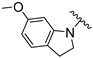	378.4	3.5	5	1	85.2	4	0.3	0.8
**3**	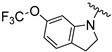	432.4	4.4	5	1	85.2	4	0.3	0.7
**4**	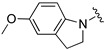	378.4	3.5	5	1	85.2	4	0.3	0.8
**5**	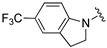	416.4	4.5	4	1	75.9	3	0.3	0.7
**6**	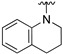	362.4	3.9	4	1	75.9	3	0.3	0.8
**7**	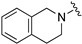	362.4	3.5	4	1	75.9	3	0.3	0.8

**Table 2:** Comparison of inhibitory activity obtained from in vitro bioassay and calculations (kcal/mol).

Compound	IC_50_ (nM) ^a^	ΔGexp ^b^	Class I		Class II	
			Gscore	BFE	Gscore	BFE
Selonsertib	0.48 ± 0.03	−12.70 ± 0.04	−8.41	−8.49 ± 0.91	−8.41	−5.50 ± 1.11
**1**	3630.00 ± 72.24	−7.41 ± 0.01	−8.26	−6.07 ± 1.24	- ^c^	- ^c^
**2**	1.55 ± 0.27	−12.02 ± 0.11	−8.11	−4.33 ± 0.82	−9.09	−9.73 ± 0.84
**3**	954.00 ± 21.51	−8.21 ± 0.01	−8.08	−5.88 ± 1.54	−8.82	−8.05 ± 1.05
**4**	45.27 ± 4.82	−10.01 ± 0.07	−8.37	−5.08 ± 1.32	−9.26	−10.01 ± 0.94
**5**	319.20 ± 10.89	−8.85 ± 0.02	−8.11	−5.45 ± 0.94	−8.97	−4.49 ± 1.01
**6**	2.92 ± 0.28	−11.64 ± 0.06	−8.50	−1.91 ± 0.69	−8.63	−7.22 ± 1.29
**7**	5112.33 ± 506.67	−7.21 ± 0.06	−8.39	−1.63 ± 1.46	−8.46	−6.31 ± 1.07

^a^ Inhibitor potency (IC_50_) as mean ± SEM (*n* = 3 experiments). ^b^ ΔG was estimated from IC_50_ values using ΔG = RTln(IC_50_), where R and T are the gas constant and the temperature (298 K), respectively. ^c^ Only one binding mode exists.

## Data Availability

The original contributions presented in this study are included in the article/Supplementary Materials. Further inquiries can be directed to the corresponding author(s).

## Supplementary Materials

The following supporting information can be downloaded at https://www.mdpi.com/article/10.3390/ijms26041527/s1.
