# Supplementary material for: Discovery of Novel Pyridin-2-yl Urea Inhibitors Targeting ASK1 Kinase and Its Binding Mode by Absolute Protein–Ligand Binding Free Energy Calculations
Source: Int J Mol Sci. 2025 Feb 12;26(4):1527. doi: 10.3390/ijms26041527 (PMC11854949; doi:10.3390/ijms26041527)
Supplement: Supplementary file 1 [file ijms-26-01527-s001.zip › ijms-3430168-supplementary.pdf]

Supporting Information for

Discovery of Novel Pyridin-2-yl Urea Inhibitors Targeting ASK1 Kinase and Its Binding Mode by Absolute Protein–Ligand Binding Free Energy Calculations

Lingzhi Wang [1,2], Yalei Gao [1,2], Yuying Chen [1,2], Zhenzhou Tang [1,2], Xiao Lin [1,2], Meng Bai [1,2], Pei Cao [1,2,*], Kai Liu [1,2,*]

[1] Guangxi Key Laboratory of Marine Drugs, Guangxi University of Chinese Medicine, Nanning 530200, China; wanglingzhi2022@stu.gxtcmu.edu.cn (L.W.); gaoyalei0322@163.com (Y.G.); ah20211209@163.com (Y.C.); tangzz@gxtcmu.edu.cn (Z.T.); linx@gxtcmu.edu.cn (X.L.); xxbai2014@163.com (M.B.)

[2] Institute of Marine Drugs, Guangxi University of Chinese Medicine, Nanning 530200, China

* Correspondence: caopeicib@hotmail.com (P.C.); kailiu@gxtcmu.edu.cn (K.L.)

CONTENTS

Table S1. Binding mode predicted by molecular docking..2

List S1. NMR information of synthesized compounds..3

Figure S1. ^{1}H NMR of Compound 2 (500 MHz, $CDCl_3$)...4

Figure S2. ^{13}C NMR of Compound 2 (126 MHz, $CDCl_3$)..4

Figure S3. ^{1}H NMR of Compound 3 (500 MHz, $CDCl_3$)...5

Figure S4. ^{13}C NMR of Compound 3 (126 MHz, $CDCl_3$)..5

Figure S5. ^{1}H NMR of Compound 4 (500 MHz, $CDCl_3$)...6

Figure S6. ^{13}C NMR of Compound 4 (126 MHz, $CDCl_3$)..6

Figure S7. ^{1}H NMR of Compound 5 (500 MHz, $CDCl_3$)...7

Figure S8. ^{13}C NMR of Compound 5 (126 MHz, $CDCl_3$)..7

Figure S9. Catalytic activity of ASK1 kinase..8

Figure S10. Inhibitory activity assay of compounds on ASK1 kinase 9

Figure S11. RMSD change of backbone and ligand under simulation (Å). 11

Figure S12. Identification of protein ASK1 purification by SDS-PAGE...................11

Table S1. Binding mode predicted by molecular docking

Compound	Class I	Class II
Selonsertib	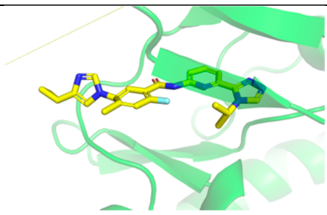 Identical to crystal 6OYT	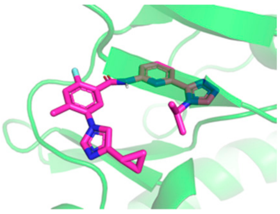
1	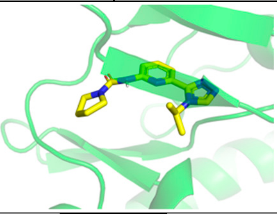	
2	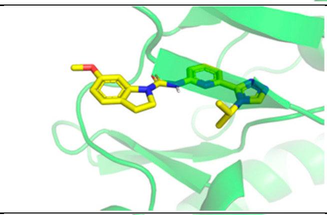	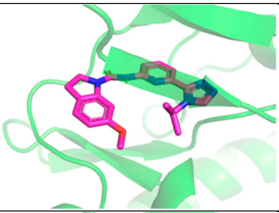
3	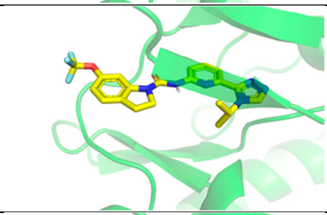	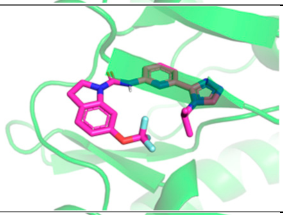
4	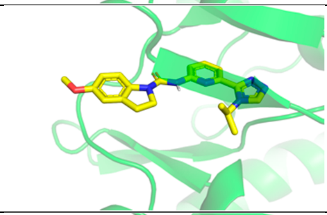	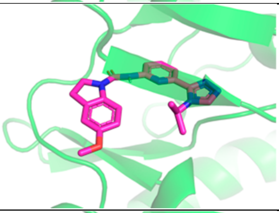
5	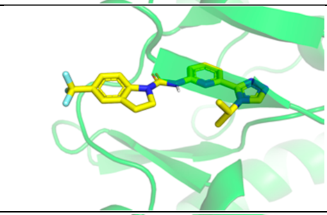	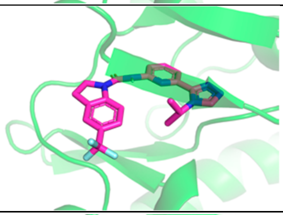
6	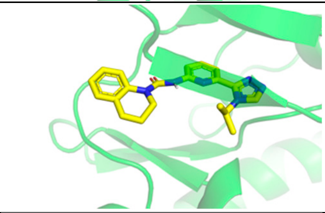	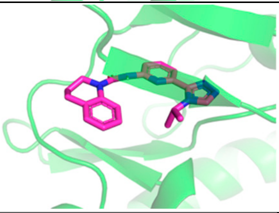
7	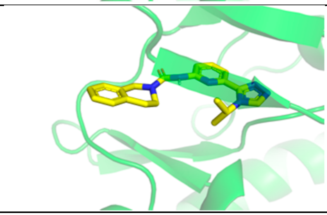	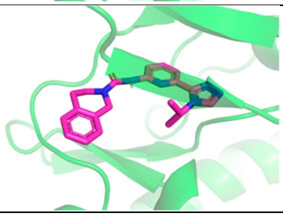

List S1. NMR information of synthesized compounds

Compound **2:**

N-(6-(4-isopropyl-4H-1,2,4-triazol-3-yl)pyridin-2-yl)-6-methoxyindoline-1-carboxamide

^{1}H NMR(500 MHz, $CDCl_3$) δ 8.35 (s, 1H), 8.17 (dd, *J* = 7.7, 1.6 Hz, 1H), 7.89 – 7.83 (m, 2H), 7.59 (d, *J* = 2.2 Hz, 1H), 7.29 (s, 1H), 7.09 (d, *J* = 8.2 Hz, 1H), 6.56 (dd, *J* = 8.1, 2.4 Hz, 1H), 5.41 (heptet, *J* = 6.7 Hz, 1H), 4.18 (t, *J* = 8.4 Hz, 2H), 3.83 (s, 3H), 3.20 (t, *J* = 8.4 Hz, 2H), 1.54 (d, *J* = 6.8 Hz, 6H).

^{13}C NMR (126 MHz, $CDCl_3$) δ 159.61, 151.22, 151.19, 150.84, 145.73, 143.67, 141.62, 139.28, 124.97, 122.59, 119.33, 113.89, 108.61, 101.42, 55.48, 48.36, 48.30, 26.96, 23.48.

HRMS(ESI): *m/z* $[M+H]^+$ calculated for $C_{20}H_{23}N_6O_2$ 379.1882, found 379.1872.

Compound **3:**

N-(6-(4-isopropyl-4H-1,2,4-triazol-3-yl)pyridin-2-yl)-6-(trifluoromethoxy)indoline-1-carboxamide

^{1}H NMR (500 MHz, $CDCl_3$) δ 8.35 (s, 1H), 8.19 – 8.14 (m, 1H), 7.91 (s, 1H), 7.86 (s, 1H), 7.28 (s, 1H), 7.18 (d, *J* = 8.1 Hz, 1H), 6.86 (dd, *J* = 8.0, 1.3 Hz, 1H), 5.36 (heptet, *J* = 6.7 Hz, 1H), 4.24 (t, *J* = 8.5 Hz, 2H), 3.29 (t, *J* = 8.5 Hz, 2H), 1.52 (d, *J* = 6.8 Hz, 6H).

^{13}C NMR (126 MHz, $CDCl_3$) δ 151.33, 151.29, 151.12, 149.02, 145.98, 144.18, 141.86, 139.60, 129.27, 125.28, 119.88, 115.44, 114.36, 109.27, 48.50, 48.37, 27.51, 23.73.

HRMS(ESI): *m/z* $[M+H]^+$ calculated for $C_{20}H_{20}F_3N_6O_2$ 433.1600, found 433.1598.

Compound **4:**

N-(6-(4-isopropyl-4H-1,2,4-triazol-3-yl)pyridin-2-yl)-5-methoxyindoline-1-carboxamide

^{1}H NMR (500 MHz, $CDCl_3$) δ 8.35 (s, 1H), 8.17 (dd, *J* = 6.4, 2.9 Hz, 1H), 7.87 – 7.79 (m, 3H), 7.21 (s, 1H), 6.80 (s, 1H), 6.76 (dd, *J* = 8.8, 2.7 Hz, 1H), 5.39 (heptet, *J* = 6.7 Hz, 1H), 4.16 (t, *J* = 8.4 Hz, 2H), 3.80 (s, 3H), 3.25 (t, *J* = 8.4 Hz, 2H), 1.54 (d, *J* = 6.8 Hz, 6H).

^{13}C NMR (126 MHz, $CDCl_3$) δ 156.29, 151.93, 151.55, 151.42, 146.17, 142.09, 139.75, 136.59, 132.90, 119.69, 115.94, 114.34, 112.56, 111.81, 56.09, 48.76, 48.18, 28.44, 24.01.

HRMS(ESI): *m/z* $[M+H]^+$ calculated for $C_{20}H_{23}N_6O_2$ 379.1882, found 379.1878.

Compound **5:**

N-(6-(4-isopropyl-4H-1,2,4-triazol-3-yl)pyridin-2-yl)-5-(trifluoromethyl)indoline-1-carboxamide

^{1}H NMR (500 MHz, $CDCl_3$) δ 8.35 (s, 1H), 8.16 (dd, *J* = 5.4, 3.9 Hz, 1H), 8.09 (d, *J* = 8.5 Hz, 1H), 7.88 – 7.83 (m, 2H), 7.50 (d, *J* = 8.5 Hz, 1H), 7.45 (s, 1H), 7.37 (s, 1H), 5.33 (heptet, *J* = 6.7 Hz, 1H), 4.27 (t, *J* = 8.6 Hz, 2H), 3.35 (t, *J* = 8.6 Hz, 2H), 1.52 (d, *J* = 6.8 Hz, 6H).

^{13}C NMR (126 MHz, $CDCl_3$) δ 151.21, 151.01, 150.89, 145.72, 141.60, 138.57, 131.08, 125.42, 125.39, 125.27, 124.90, 124.64, 123.12, 121.69, 121.66, 119.67, 115.00, 114.17, 48.23, 47.71, 27.47, 23.48.

HRMS(ESI): *m/z* $[M+H]^+$ calculated for $C_{20}H_{20}F_3N_6O$ 417.1651, found 417.1646.

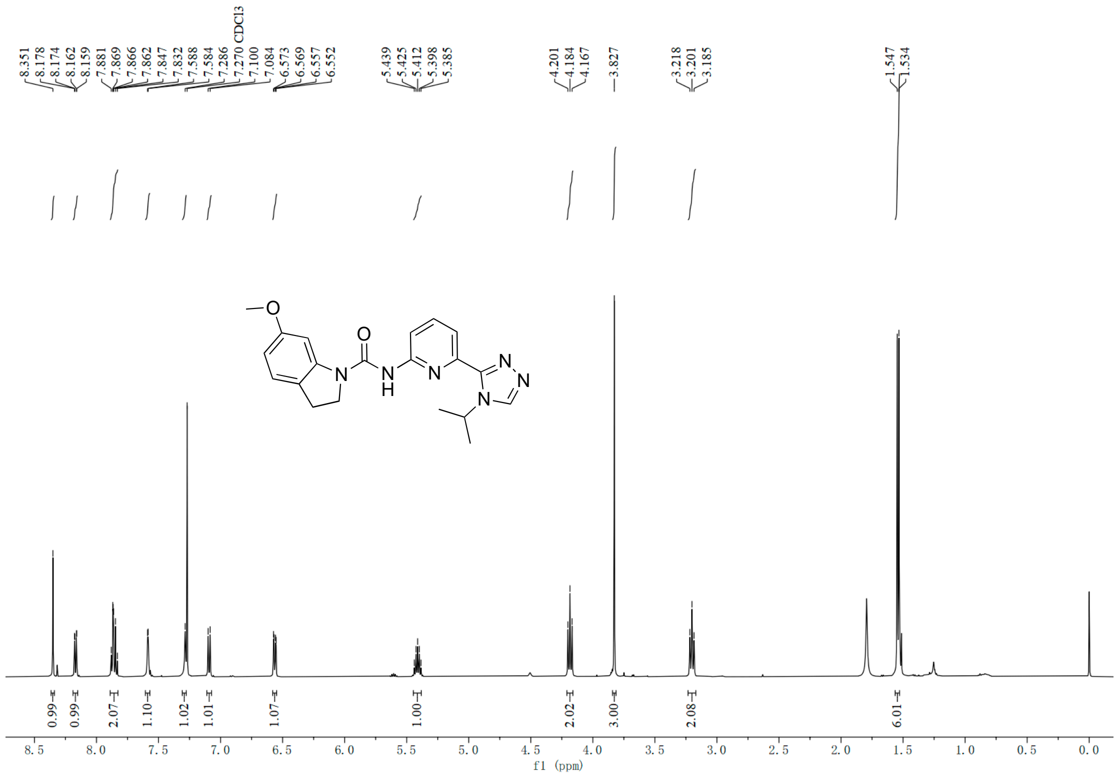

Figure S1. ^{1}H NMR of Compound 2 (500 MHz, $CDCl_3$)

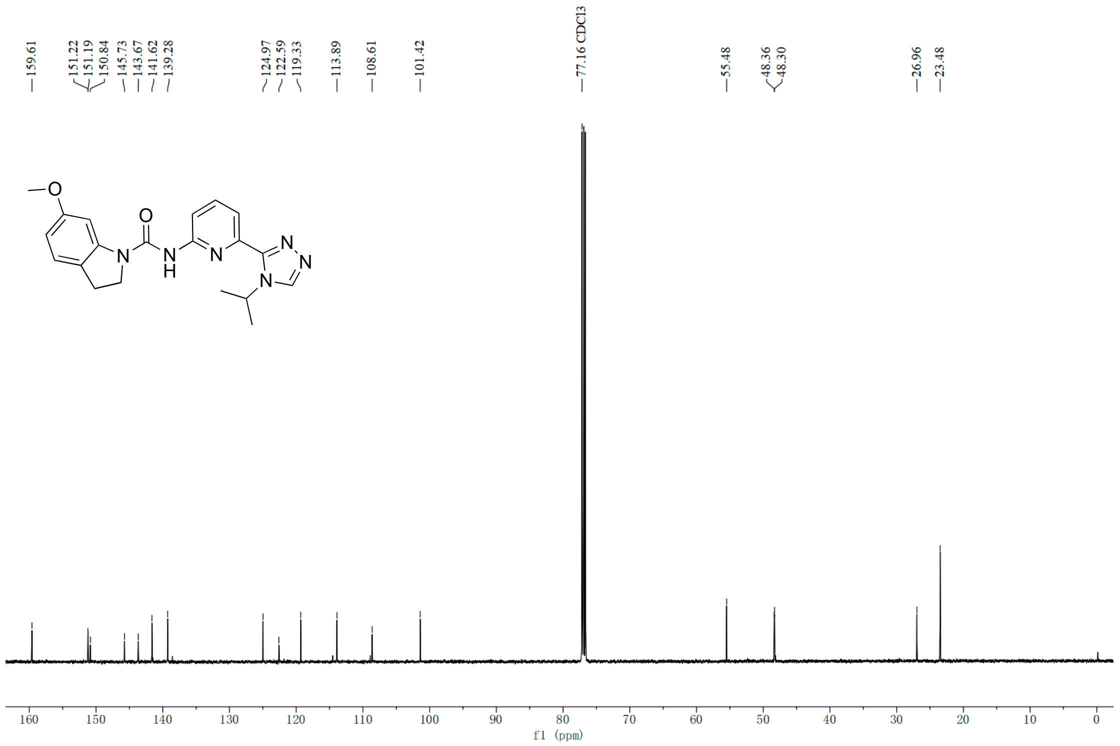

Figure S2. ^{13}C NMR of Compound 2 (126 MHz, $CDCl_3$)

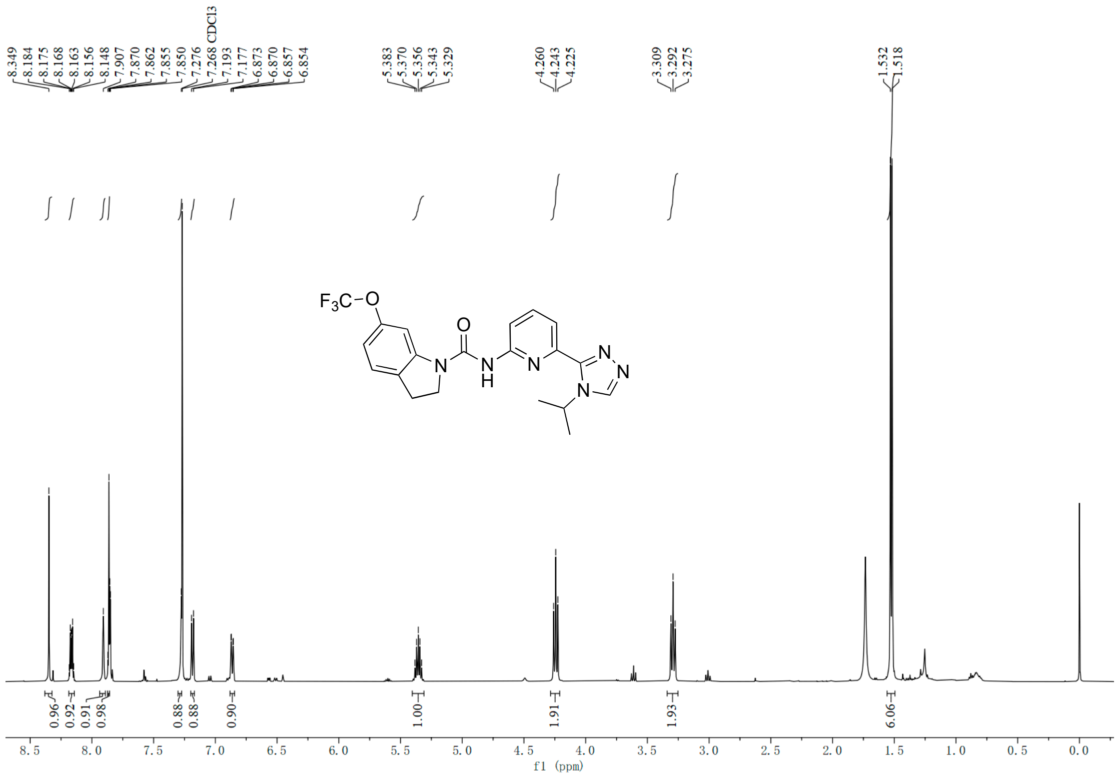

Figure S3. ^{1}H NMR of Compound 3 (500 MHz, $CDCl_3$)

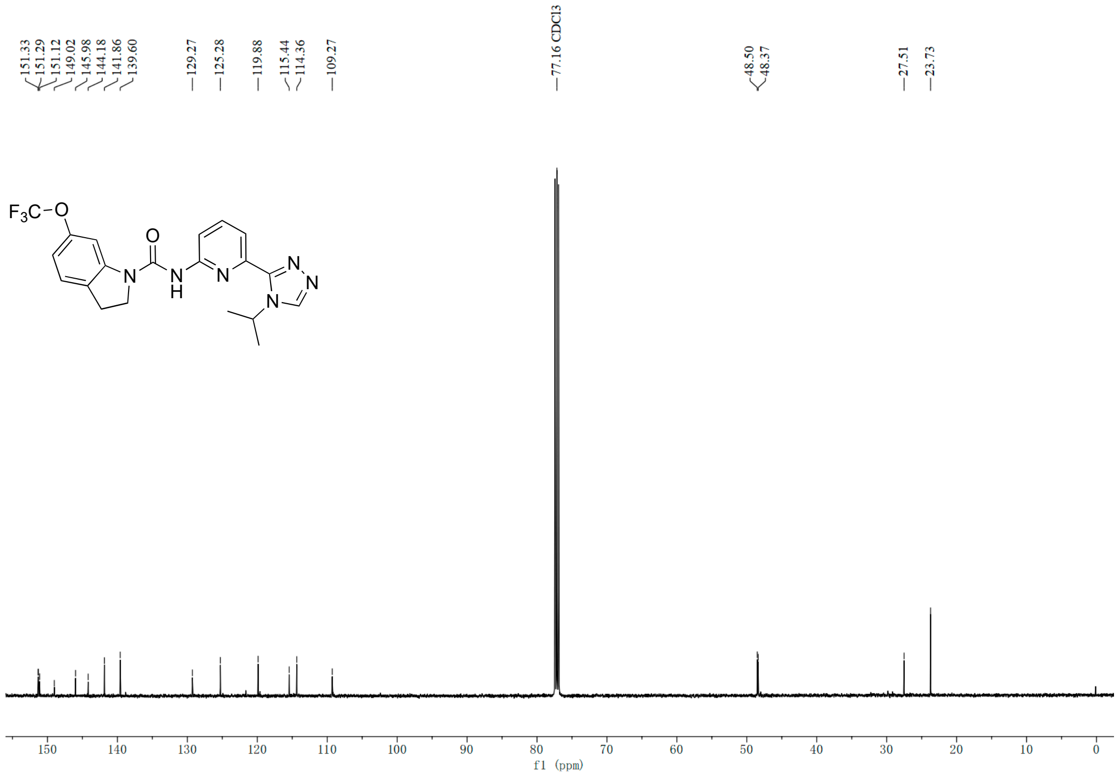

Figure S4. ^{13}C NMR of Compound 3 (126 MHz, $CDCl_3$)

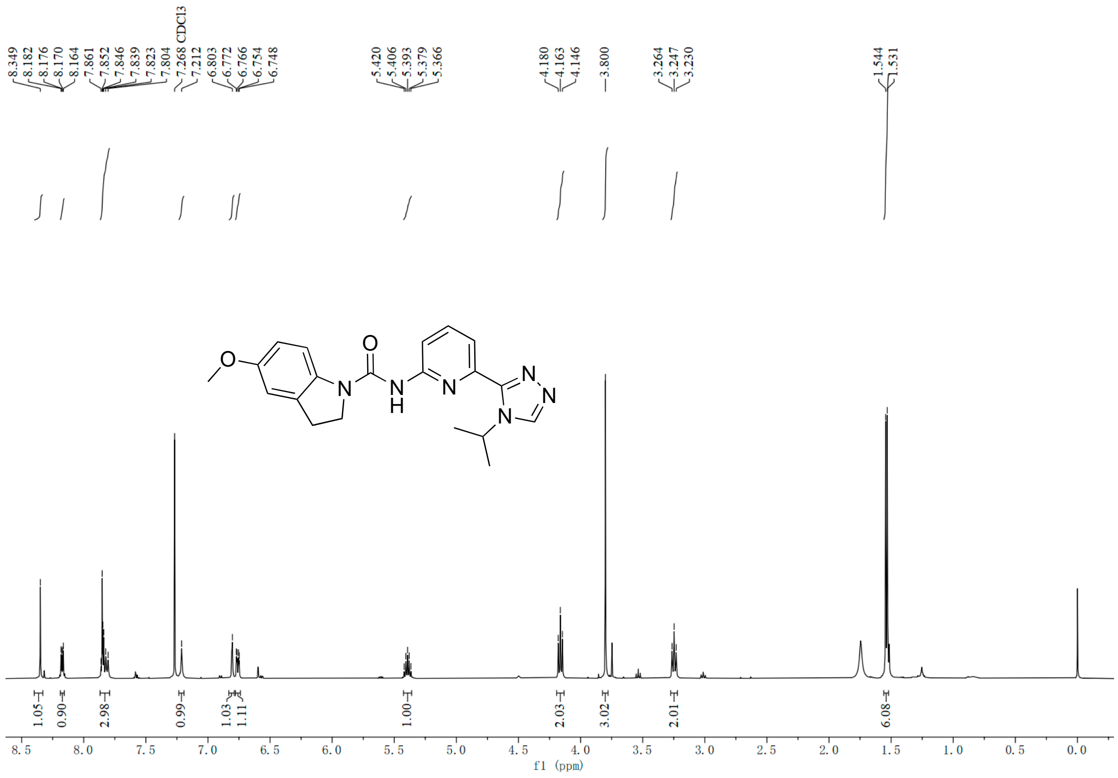

Figure S5. ^{1}H NMR of Compound 4 (500 MHz, $CDCl_3$)

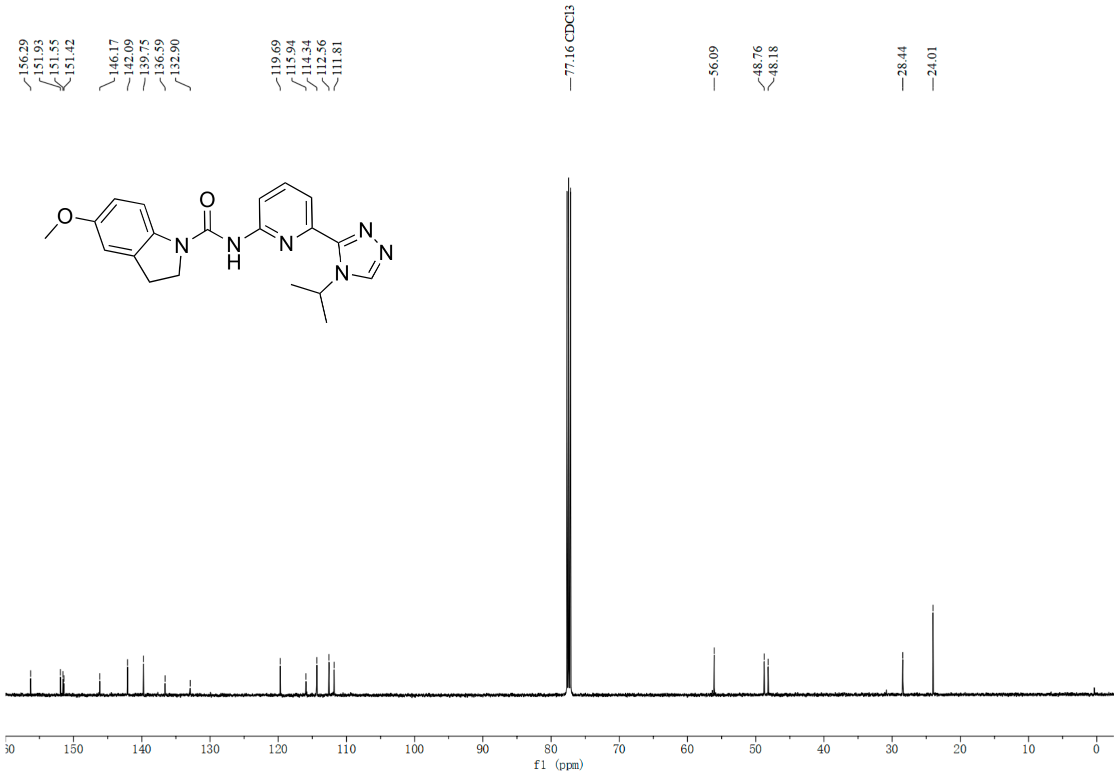

Figure S6. ^{13}C NMR of Compound 4 (126 MHz, $CDCl_3$)

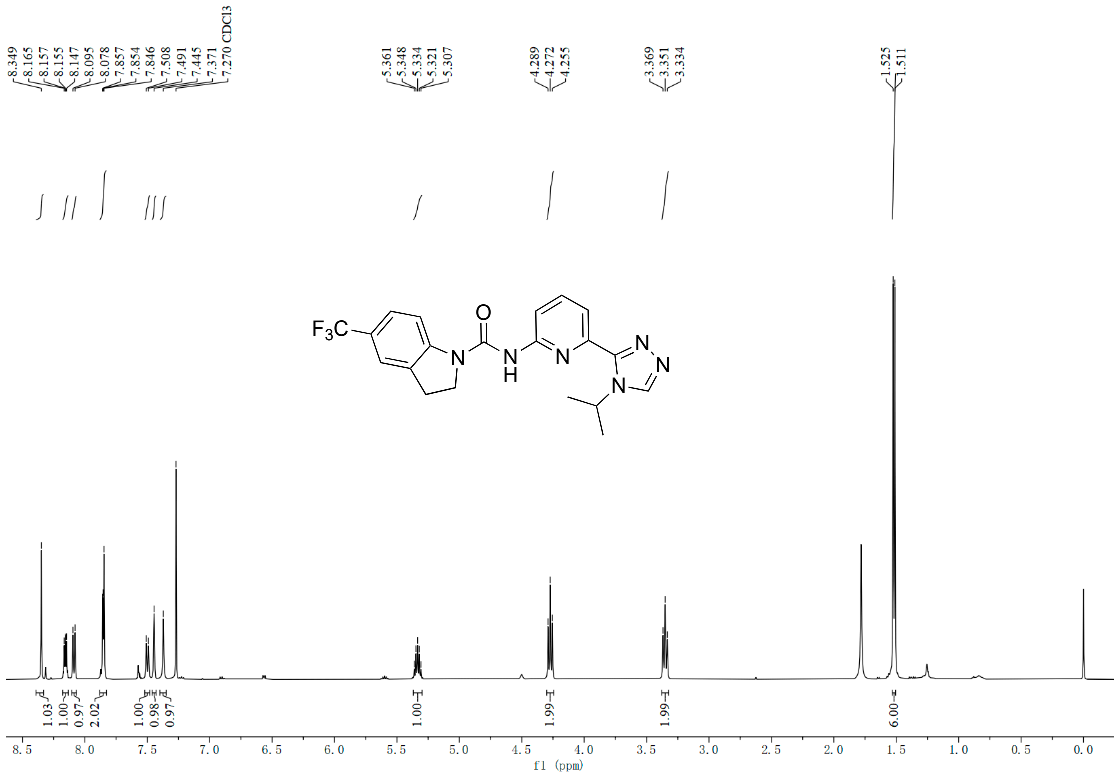

Figure S7. ^{1}H NMR of Compound 5 (500 MHz, $CDCl_3$)

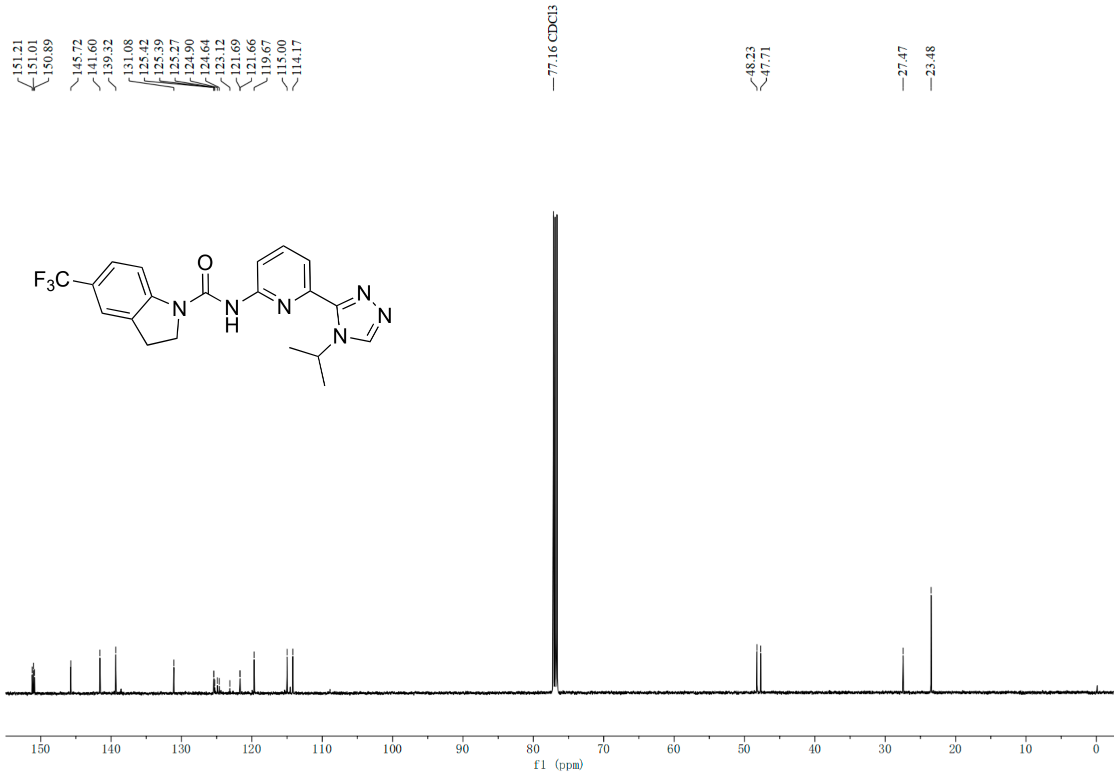

Figure S8. ^{13}C NMR of Compound 5 (126 MHz, $CDCl_3$)

Protein	EC_{50} (ng)	Replica1	Replica2	Replica3
ASK1	41.60±0.55	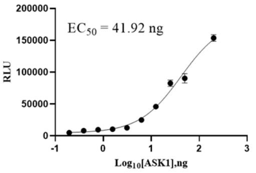	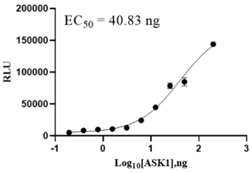	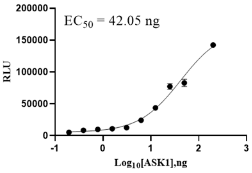

Figure S9. Catalytic activity of ASK1 kinase

Compound	IC_{50} (nM)	Replica1	Replica2	Replica3
Selonsertib	0.48±0.03	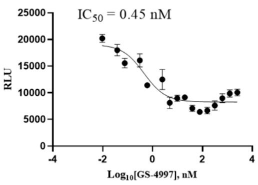	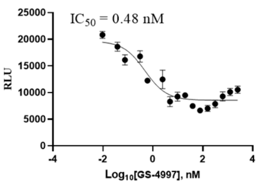	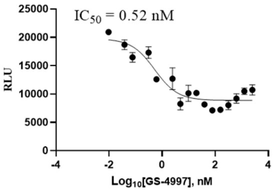
1	3630.00±72.24	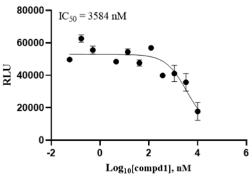	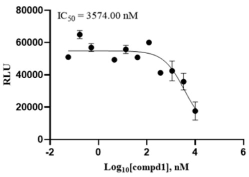	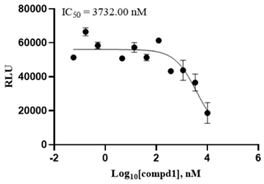
2	1.55±0.27	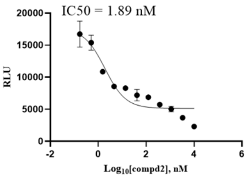	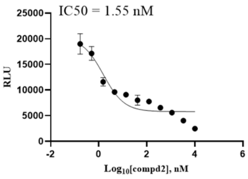	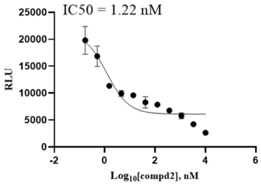
3	954.00±21.51	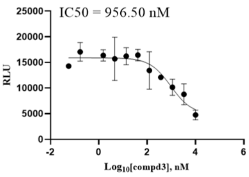	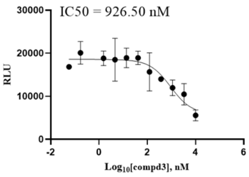	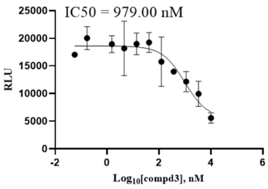
4	45.27±4.82	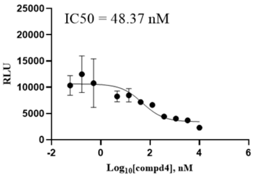	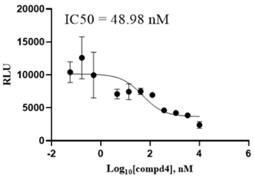	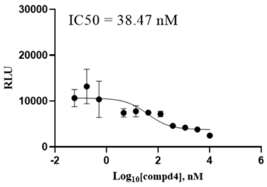
5	319.20±10.89	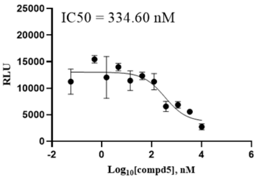	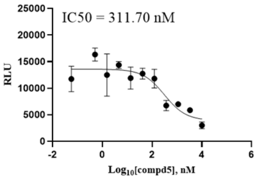	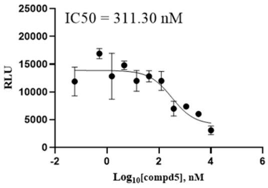

6	2.92±0.28	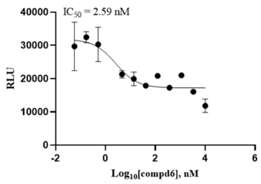	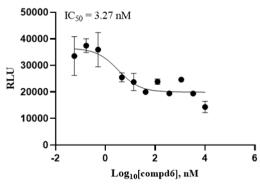	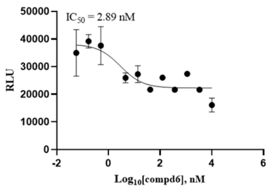
7	5112.33±506.67	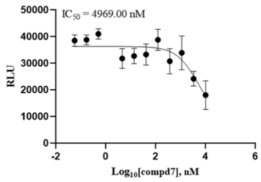	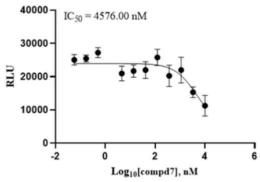	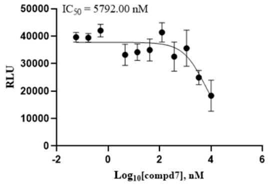

Figure S10. Inhibitory activity assay of compounds on ASK1 kinase

**Compound**	**Class I**	**Class II**
Selonsertib	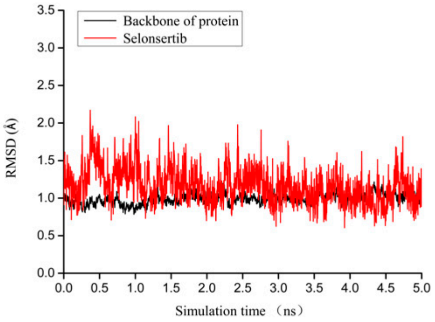	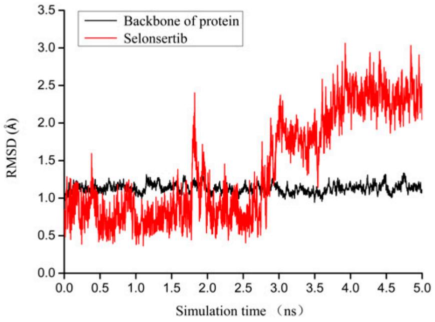
**1**	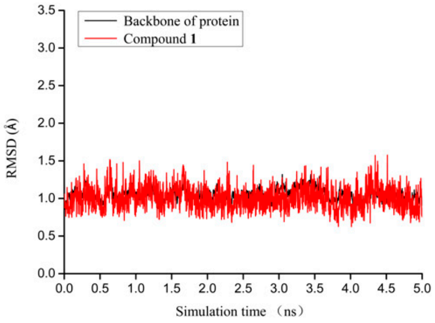	
**2**	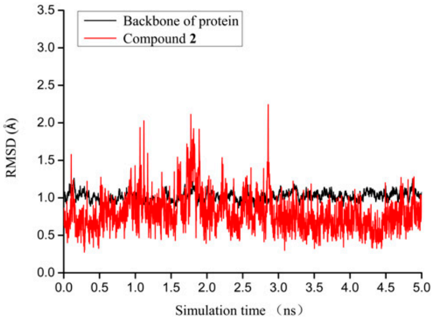	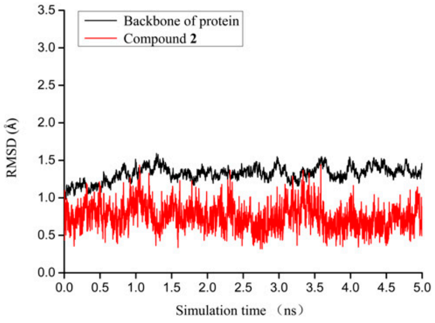

**3**	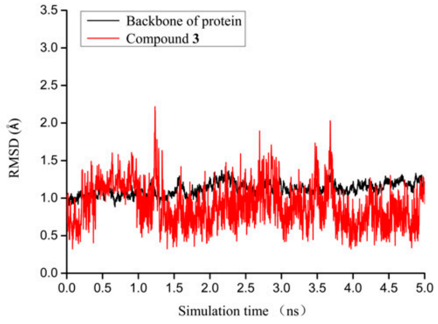Backbone of protein<br>Compound 3<br>RMSD (Å)<br>Simulation time （ns）	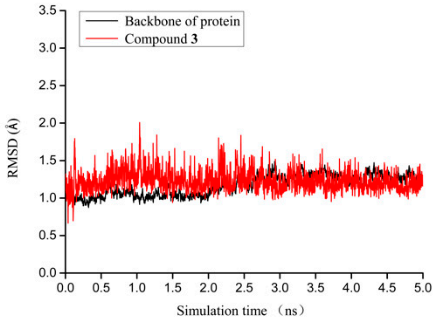Backbone of protein<br>Compound 3<br>RMSD (Å)<br>Simulation time （ns）
**4**	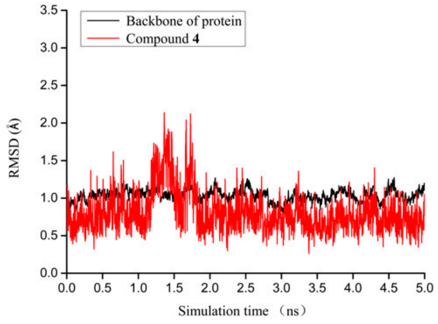Backbone of protein<br>Compound 4<br>RMSD (Å)<br>Simulation time （ns）	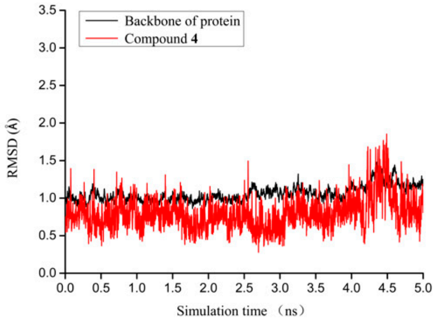Backbone of protein<br>Compound 4<br>RMSD (Å)<br>Simulation time （ns）
**5**	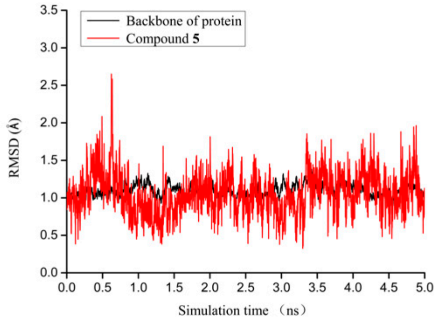Backbone of protein<br>Compound 5<br>RMSD (Å)<br>Simulation time （ns）	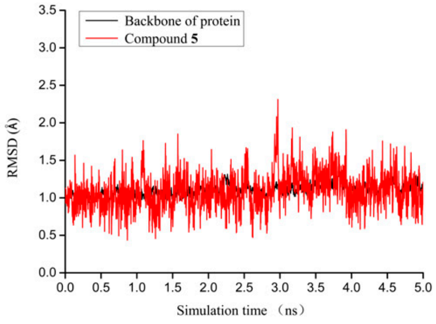Backbone of protein<br>Compound 5<br>RMSD (Å)<br>Simulation time （ns）
**6**	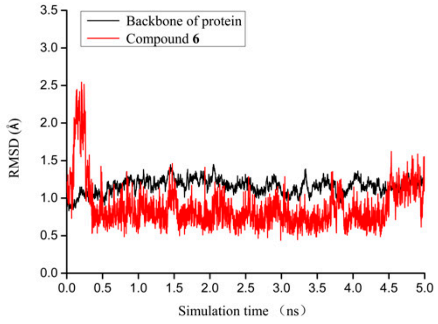Backbone of protein<br>Compound 6<br>RMSD (Å)<br>Simulation time （ns）	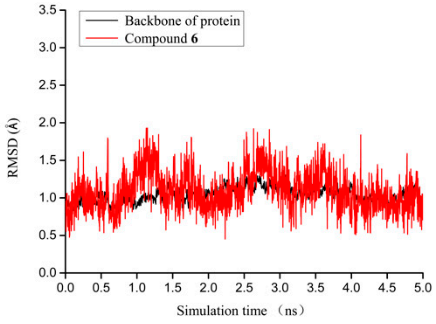Backbone of protein<br>Compound 6<br>RMSD (Å)<br>Simulation time （ns）

7

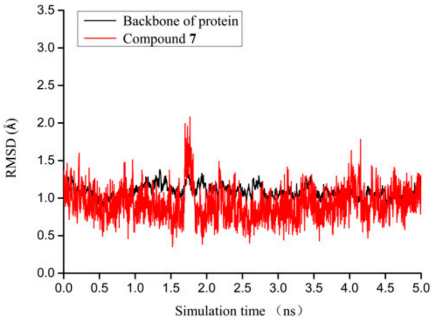

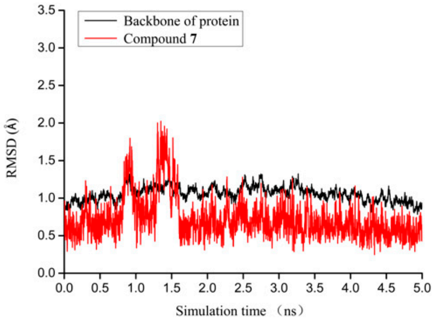

Figure S11. RMSD change of backbone and ligand under simulation (Å).

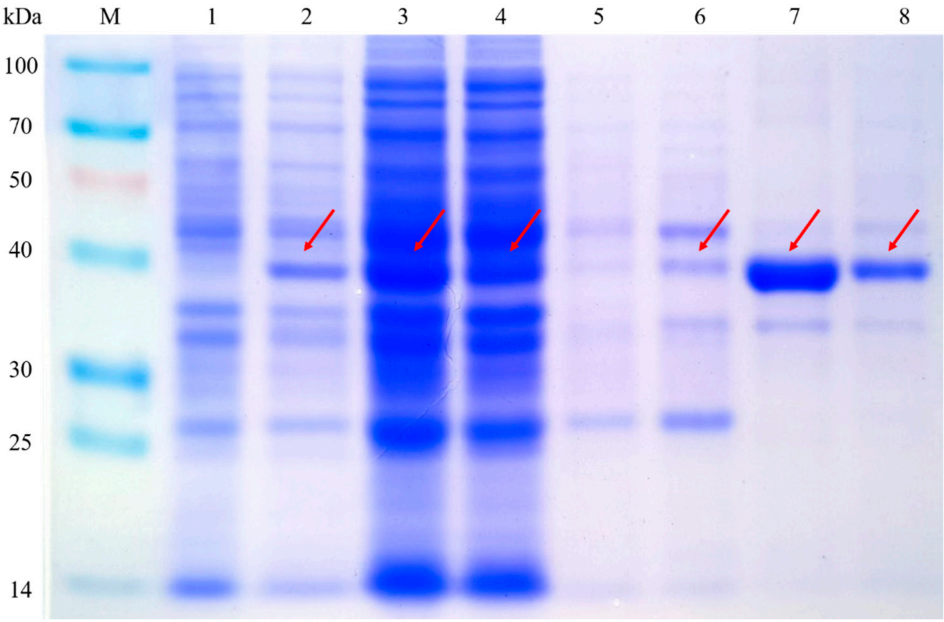

Figure S12. Identification of protein ASK1 purification by SDS-PAGE.

(Note: M: protein molecular weight standard; Lane 1: Control of un-induced supernatant; Lane 2: Control of induced supernatant; Lane 3: Supernatant after ultrasound; Lane 4: Flow-through liquid; Lanes 5-8: Elution with 25, 125, 250 and 500 mmol/L imidazole. The arrows indicate the target protein, ASK1.)
